# Correction: “Actually, even me I wouldn’t think that it is there” exploring the knowledge and attitudes of health professionals towards autism spectrum disorders in Uganda

**DOI:** 10.1371/journal.pmen.0000587

**Published:** 2026-03-26

**Authors:** Rosco Kasujja, Barbra Yeko Kissa, Tara Murphy, Kirstie Fleetwood-Meade

The second author’s name is spelled incorrectly. The correct name is: Barbra Yeko Kissa.
