# Supplementary material for: “Actually, even me I wouldn’t think that it is there” exploring the knowledge and attitudes of health professionals towards autism spectrum disorders in Uganda
Source: PLOS Ment Health. 2024 Jul 30;1(2):e0000088. doi: 10.1371/journal.pmen.0000088 (PMC12798202; doi:10.1371/journal.pmen.0000088)
Supplement: Attachment — Submitted filename: Responses TM.docx [file pmen.0000088.s001.docx]

Responses to the comments raised by the various Reviewers.

| # | Reviewer’s comments | Response |
| --- | --- | --- |
| 1. | The title is very long and difficult to comprehend the first section. This part can be deleted and simply state "Exploring the knowledge and attitudes of health professionals towards Autism Spectrum Disorder in Uganda" | We appreciate your suggestion to simplify the title for clarity. We have revised the title to: "**Exploring the Knowledge and Attitudes of Health Professionals Towards Autism Spectrum Disorder in Uganda**." We believe that the new title retains the essential elements of the study. (pg 1) |
| 2. | The study aimed to explore knowledge and attitudes, why did the authors leave out practices as is commonly done in KAP (Knowledge, Attitude and Practices) studies? | You raised a pertinent point about the omission of practices in our study, which are typically included in KAP (Knowledge, Attitude, and Practices) studies. We chose to focus specifically on knowledge and attitudes due to the exploratory nature of this initial study and the limited scope of our resources. We acknowledge that incorporating practices would provide a more comprehensive understanding. Future research could indeed adopt the full KAP model to facilitate comparisons with other studies. We now include this suggestion in the Discussion section (pg 19). |
| 3. | How was the sample size of 20 participants determined? Was it the point of saturation? | The sample size of 20 participants was determined based on the principle of data saturation, which we achieved during our interviews. Saturation was assessed through iterative analysis, ensuring that no new themes were emerging as we approached the final interviews. We now clarify this in the Methods section to ensure accuracy.  We have added “The sample size of 20 was arrived at one saturation had been reached and no new themes emerged in the interviews." (pg 4) |
| 4. | Page 10 sentence 3 is incomplete "...ASD are not brought for care due to___________" | Thank you for pointing out the incomplete sentence on page 10. We have corrected it to read: "...ASD are not brought for care due to the lack of awareness and understanding among caregivers and the community (pag 7) |
| 5. | The section for "Education implication" is not clear. The authors need to check the tense used at the beginning of this section. It implies that the study involved educationists. | We acknowledge the confusion caused by the tense used in the "Education Implication" section and the implication that the study involved educationists. We have revised this section for clarity and consistency in tense (pg 17) |
| 6. | In the conclusion, the authors could suggest future direction of research. | Added, thank you. (pg 19) |
| 7. | Areas for Improvement: Enhancements such as broadening the sample size, integrating quantitative research methods, and possibly diversifying the range of healthcare settings would significantly improve the manuscript. These modifications could deepen the study's impact and merit consideration for resubmission and publication. | This study took a qualitative approach and focused on qualitative data. |
| 8. | The manuscript does not explicitly state its adherence to these specific guidelines. Nonetheless, reviewing and aligning with guidelines like STROBE, which is pertinent for observational studies, would bolster the manuscript's credibility and reflect adherence to the highest standards of research reporting. | We appreciate this observation. However, upon careful review, it is evident that this manuscript adheres to the STROBE guidelines, encompassing all the essential items outlined in the checklist. |
| 9. | The manuscript is effectively organized and articulately composed, ensuring accessibility to a broad audience. Expanding upon the implications of the findings for healthcare policy and practice, and including a more detailed discussion on practical applications, would further enhance its relevance and utility to both specialists and non-specialists. | Thank you for pointing out this. These parts have been improved. |
| 10. | The manuscript is effectively organised and articulately composed, ensuring accessibility to a broad audience. Expanding upon the implications of the findings for healthcare policy and practice, and including a more detailed discussion on practical applications, would further enhance its relevance and utility to both specialists and non-specialists. | The implications have been added (pg 17) |
